# Pretreatment carcinoembryonic antigen level is a risk factor for para-aortic lymph node recurrence in addition to squamous cell carcinoma antigen following definitive concurrent chemoradiotherapy for squamous cell carcinoma of the uterine cervix

**DOI:** 10.1186/1748-717X-7-13

**Published:** 2012-01-30

**Authors:** Eng-Yen Huang, Yu-Jie Huang, Chan-Chao Chanchien, Hao Lin, Chong-Jong Wang, Li-Min Sun, Chin-Wen Tseng, Ching-Chou Tsai, Yu-Che Ou, Hung-Chun Fu, Hui-Chun Chen, Hsuan-Chih Hsu, Chang-Yu Wang

**Affiliations:** 1 Department of Radiation Oncology, Kaohsiung Chang Gung Memorial Hospital, Chang Gung University College of Medicine, Kaohsiung, Taiwan; 2 Department of Gynecologic Oncology, Kaohsiung Chang Gung Memorial Hospital, Chang Gung University College of Medicine, Kaohsiung, Taiwan; 3 School of Traditional Chinese Medicine, Chang Gung University College of Medicine, Taoyuan, Taiwan; 4 School of Medicine, Chang Gung University College of Medicine, Taoyuan, Taiwan; 5 Department of Radiation Oncology, Zuoying Armed Forces General Hospital, Kaohsiung, Taiwan; 6 Department of Gynecologic Oncology, Chia-Yi Chang Gung Memorial Hospital, Chia-Yi, Taiwan

**Keywords:** carcinoembryonic antigen, para-aortic lymph node, squamous cell carcinoma, uterine cervix, SCC-Ag, radiotherapy

## Abstract

**Background:**

To identify pretreatment carcinoembryonic antigen (CEA) levels as a risk factor for para-aortic lymph node (PALN) recurrence following concurrent chemoradiotherapy (CCRT) for cervical cancer.

**Methods:**

From March 1995 to January 2008, 188 patients with squamous cell carcinoma (SCC) of the uterine cervix were analyzed retrospectively. No patient received PALN irradiation as the initial treatment. CEA and squamous cell carcinoma antigen (SCC-Ag) were measured before and after radiotherapy. PALN recurrence was detected by computer tomography (CT) scans. We analyzed the actuarial rates of PALN recurrence by using Kaplan-Meier curves. Multivariate analyses were carried out with Cox regression models. We stratified the risk groups based on the hazard ratios (HR).

**Results:**

Both pretreatment CEA levels ≥ 10 ng/mL and SCC-Ag levels < 10 ng/mL (*p *< 0.001, HR = 8.838), SCC-Ag levels ≥ 40 ng/mL (*p *< 0.001, HR = 12.551), and SCC-Ag levels of 10-40 ng/mL (*p *< 0.001, HR = 4.2464) were significant factors for PALN recurrence. The corresponding 5-year PALN recurrence rates were 51.5%, 84.8%, and 27.5%, respectively. The 5-year PALN recurrence rate for patients with both low (< 10 ng/mL) SCC and CEA was only 9.6%. CEA levels ≥ 10 ng/mL or SCC-Ag levels ≥ 10 ng/mL at PALN recurrence were associated with overall survival after an isolated PALN recurrence. Pretreatment CEA levels ≥ 10 ng/mL were also associated with survival after an isolated PALN recurrence.

**Conclusions:**

Pretreatment CEA ≥ 10 ng/mL is an additional risk factor of PALN relapse following definitive CCRT for SCC of the uterine cervix in patients with pretreatment SCC-Ag levels < 10 ng/mL. More comprehensive examinations before CCRT and intensive follow-up schedules are suggested for early detection and salvage in patients with SCC-Ag or CEA levels ≥ 10 ng/mL.

## Background

Although concurrent chemoradiotherapy (CCRT) is a standard treatment for locally advanced cervical cancer [1,2], para-aortic lymph node (PALN) recurrence was not uncommon in our prior study, demonstrating that pelvic CCRT did not decrease PALN recurrence more than pelvic RT alone [3]. Hence, the strategy to reduce PALN recurrence is an important issue for the treatment of locally advanced cervical cancer. In addition to stage, squamous cell carcinoma antigen (SCC-Ag) is an important tumor marker in the prediction of overall survival [4], disease-specific survival [4], and distant metastasis [5] in patients undergoing RT alone. We first identified SCC-Ag as a significant factor of PALN recurrence in patients treated with definitive radiotherapy or CCRT [3]. However, the impact of the other tumor marker, carcinoembryonic antigen (CEA), in PALN recurrence is not clear in patients undergoing pelvic CCRT. In our clinical practice, CEA may be a potential risk factor for PALN recurrence in addition to SCC-Ag.

The aim of this retrospective study is to identify CEA as a risk factor for PALN recurrence following definitive CCRT for squamous cell carcinoma of the cervix. We performed risk stratification of PALN recurrence based on levels of tumor markers, and we recommend comprehensive detection of PALN metastasis or intensive surveillance of patients at risk of PALN recurrence, especially those patients with both high CEA and low SCC-Ag levels.

## Methods

### Patients' characteristics

Between March 1995 and January 2008, we retrospectively reviewed 246 consecutive patients with histologically proven squamous cell carcinoma of the cervix undergoing CCRT. These patients had an Eastern Cooperative Oncology Group (ECOG) performance status of 0-2, Federation Internationale de Gynecologie et d'Obstetrique (FIGO) Stage IB-IVA without PALN metastasis on a CT scan, and an intact uterus. For the purpose of this study, we excluded 19 patients without a pretreatment CEA/SCC-Ag measurement and 39 patients without an abdominal CT scan examination during follow-up. Finally, 188 patients were included in this review. The characteristics of these patients are shown in Table 1. Prior to radiotherapy, patients were evaluated by physical examination, routine laboratory tests (such as CEA, SCC-Ag, complete blood count, creatinine, and blood urea nitrogen), abdominal-pelvic computed tomography (CT) scan, and chest x-ray. No patient received a PET scan examination at diagnosis. Scoring for parametrial (PM) involvement was based on the concept of tumor burden in previous studies [6,7].

**Table 1:** Patient characteristics (n = 188)

Parameters	No. (%)	Mean ± SEM
Age (years)		56.5 ± 0.8
Stage		
I	22 (11.7%)	
II	125 (66.5%)	
III	37 (19.7%)	
IV	4 (2.1%)	
Smoking		
No	173 (92.0%)	
Yes	15 (8.0%)	
Pelvic lymphadenopathy on CT scan		
No	167 (88.8%)	
Yes	21 (11.2%)	
Parametrial scores		
0	36 (19.1%)	
1-3	103 (54.8%)	
4-6	49 (26.1%)	
Hemoglobin (g/dL)		11.1 ± 0.2
Field size (Y-axis) (cm)		18.3 ± 0.1
HDR dose to point A (Gy)		24.3 ± 0.2
RT duration (days)		61.2 ± 1.1
Pretreatment SCC-Ag level (ng/mL)		15.2 ± 2.3
< 2	60 (31.9%)	
2-10	68 (36.2%)	
10-20	21 (11.6%)	
20-40	21 (11.6%)	
≥ 40	18 (9.6%)	
Pretreatment CEA level (ng/mL)		7.7 ± 1.7
< 5	149 (79.3%)	
5-10	16 (8.5%)	
≥ 10	23 (12.2%)	
EBRT technique		
AP/PA	2 (1.1%)	
Four-field	174 (92.6%)	
IMRT	12 (6.4%)	
EBRT dose (Gy)		
Central dose		42.2 ± 0.3
Parametrial dose		47.2 ± 0.4

The institutional review board of our Hospital approved the present study (99-2005B).

### External beam radiation therapy (EBRT)

All patients initially received external beam radiation therapy (EBRT) of the pelvis. Typically, the four-field technique was used for orthogonal films or a CT-based simulation. In patients with anteroposterior/posteroanterior (AP/PA) or four-field irradiation, the whole-pelvic (WP) fields were arranged as follows. The upper border of the AP/PA fields was located at the L4-5 passage. The lateral border was 1.5-2 cm beyond the widest part of the pelvic brim. For the bilateral fields, the anterior border was the anterior part of the pubic symphysis. The posterior border was at least behind the S2-3 passage. Modification of the WP fields may depend on the physician's decisions about such factors as old age and a lower small-bowel position. In 12 patients undergoing IMRT, the clinical target volume (CTV) included the gross tumor, uterus, parametrium, pelvic side wall, regional lymph nodes (presacral, common iliac, and internal iliac and external iliac), and vagina. The planning target volume (PTV) was expanded 10 mm from CTV. IMRT was planned (CadPlan, Subiaco, Australia) and delivered through 5 to 7 fields by 6 MV photons. The dose per fraction was 1.8 Gy, 5 fractions weekly. The planning dose was 39.6-45 Gy/22-25 fractions for the initial pelvic fields. An additional boost (5.4-18 Gy/3-10 fractions) was given to the bilateral PM or low pelvis in patients with cervical cancer advanced beyond Stage IIA. Monthly concurrent cisplatin chemotherapy and 5-FU-based concurrent chemotherapy for 1-8 courses (median 3) was used in all patients. The typical dose of cisplatin was 50 mg/m^2^, followed by 5-FU 4 gm through a 96-hour infusion.

### High dose-rate intracavitary brachytherapy (HDR-ICBT)

After completion of EBRT, patients underwent high-dose-rate intracavitary brachytherapy (HDR-ICBT) that delivered a boost of radiation to the cervical tumor through a remote afterloading system (microSelectron, Nucletron Co., The Netherlands). The isotope was ^192^Ir sources. The applicator was the Henschke's type. Before applicator implantation, patients received local vaginal application of lidocaine spray and/or intramuscular pethidine injections for anesthesia. After dilatation of the cervical canal, the applicator was inserted into the uterine cavity and bilateral fornix. The vaginal canal was packed with gauze to spare it from the high dose of radiation in the bladder and rectum. Orthogonal films were taken for dose calculation in the planning system (TheraPlan 500, Theratronics Co., Canada; Nucletron PLATO-RTS version 2.0, The Netherlands). The dose optimization was described in our previous study [8]. The prescribed dose was 3.5-6 Gy at Point A for 4-6 fractions (median 5), and was delivered twice per week; the exact dose and fractions depended on the physician's decision and the patient's condition.

### Tumor markers measurement

Measurement of CEA and SCC-Ag were performed by radioimmunoassay. After 2005, the method of SCC-Ag assay was changed to electrochemiluminescence immunoassay. The pretreatment measurement was 1-2 weeks before CCRT. The frequency of tumor markers measurement during CCRT was dependent on physician's preference. They were checked usually in the completion of EBRT and ICBT. Although tumor markers were routinely checked after complete of CCRT, the variation of frequency and interval was based on physician's preference, interval of follow-up, and disease status. Once treatment failure such as PALN recurrence, distant metastasis, or locoregional recurrence was detected, the tumor markers were checked simultaneously for additional information.

### Follow-up

After the completion of radiotherapy, follow-ups of patients were scheduled in the department of Radiation and/or Gynecological oncology. Laboratory tests, chest x-ray, physical examination, and CT scan examination were carried out. A radiologist diagnosed PALN recurrence using an abdominal CT scan. Isolated PALN recurrence was defined as the first site of recurrence without concurrent locoregional or distant failure. Non-isolated PALN recurrence was defined as PALN recurrence along with other failure(s). For example, PALN recurrence with coexisting or following distant organ metastasis or locoregional recurrence. The PALN recurrence-free interval was calculated from the completion of radiotherapy to the last CT scan examination showing no evidence of PALN recurrence with or without distant organ metastasis.

### Statistics

The univariate analysis of PALN recurrence was carried out by the Kaplan-Meier method, and the statistical significance of the different groups was tested by the log-rank test. We used the Cox proportional hazards model with the stepwise forward procedure for multivariate analysis. We input the following variables into a regression model. All variables such as age, stage, PM score, tumor size, pelvic lymphadenopathy, radiation field size, smoking, hemoglobin level, pretreatment CEA, and SCC-Ag level for Cox regression were considered as categorical data. A *p *value < 0.05 was statistically significant. The relative risk of PALN recurrence was represented by the hazard ratio (HR) with a 95% confidence interval (CI) in the Cox regression. Data processing and statistics were carried out on a personal computer using the SPSS 17.0 software for Windows (SPSS Inc., Chicago, USA).

## Results

### Outcome of PALN recurrence

The median time of follow-up was 58.2 months (range 3.6-178.2). Following radiotherapy, 35 patients (18.6%) had PALN recurrence. There were 19 patients (8.6%) with isolated PALN recurrence. PALN recurrence was detected by routine CT scan examination in 7 patients (20%) without increased tumor markers or symptoms. The pretreatment SCC-Ag levels of isolated and non-isolated PALN recurrence were 16.7 ± 4.2 and 45.4 ± 16.7 ng/mL, respectively. The corresponding CEA levels were 5.2 ± 1.3 and 25.1 ± 14.8 ng/mL.

**Figure 1:**
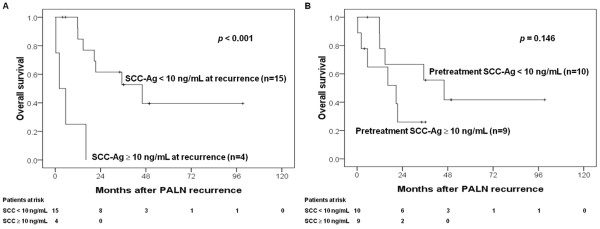
**(A) SCC-Ag ≥ 10 ng/mL at recurrence but not (B) pretreatment SCC-Ag levels predicted overall survival after isolated PALN recurrence**.

**Figure 2:**
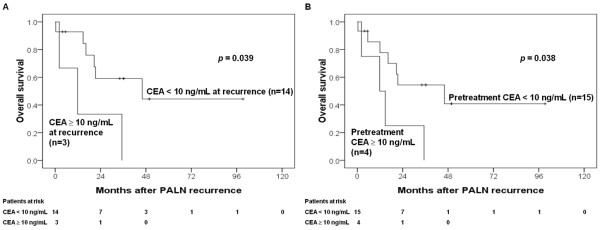
**(A) CEA ≥ 10 ng/mL at recurrence and (B) pretreatment CEA levels predicted overall survival after isolated PALN recurrence**.

The 5-year actuarial PALN recurrence rates were 23.8%. No further treatment was noted in 13 patients. At the time, 12, 1, and 9 patients were undergoing radiotherapy plus chemotherapy, radiotherapy, and chemotherapy for salvage, respectively. The 3-year overall survival rates after non- isolated PALN recurrence were 7%. The corresponding rates of isolated PALN recurrence were 40.9%. The 3-year survival rates in patients with isolated recurrence were 52.7% and 0% (*p *< 0.001) at SCC-Ag levels < 10 and ≥ 10 ng/mL of isolated PALN recurrence (Figure 1A), respectively. However, pretreatment SCC-Ag levels did not affect survival rates after isolated PALN recurrence (*p *= 0.146) (Figure 1B). Both CEA levels ≥ 10 ng/mL (*p *= 0.039) at PALN recurrence (Figure 2A) and pretreatment CEA levels ≥ 10 ng/mL (*p *= 0.038) (Figure 2B) were prognostic factors of overall survival following isolated PALN recurrence. The 3-year survival rate was 64.8% in patients undergoing salvage CCRT for isolated PALN recurrence.

### Multivariate analyses of PALN recurrence

We performed multivariate analysis for factors predictive of PALN recurrence (Table 2). CEA levels ≥ 10 ng/mL (*p *= 0.001), SCC-Ag levels 10-40 ng/mL (*p *= 0.018), SCC-Ag levels ≥ 40 ng/mL (*p *< 0.001) were significant factors in the first Cox regression. Based on our prior study [9], CEA can interact with SCC-Ag levels for prognosis. We performed a second Cox regression considering the interaction between CEA levels and SCC-Ag levels and noted the interaction (*p *< 0.001). Hence, we categorized the new tumor marker groups as CEA levels < 10 ng/mL and ≥ 10 ng/mL in patients with SCC levels < 10 ng/mL. The remaining groups of SCC-Ag levels were 10-40 ng/mL and ≥ 40 ng/mL. We performed the third Cox regression (Table 3) and noted that both CEA ≥ 10 ng/mL and SCC-Ag levels < 10 ng/mL (*p *< 0.001) and SCC-Ag levels ≥ 40 ng/mL (*p *< 0.001), and SCC-Ag levels of 10-40 ng/mL (*p = *0.002) were significant factors.

**Table 2:** Multivariate analysis of PALN recurrence

Parameters	Number	Hazard ratio (95% CI)	*p *value
Age			0.233
< 50	58	reference	
50-60	59	--	0.124
> 60	71	--	0.141
Stage			0.831
I	22	reference	
II	145	--	0.852
III-IVA	41	--	0.686
Parametrial score			0.056
0	36	reference	
1-3	103	--	0.749
4-6	49	--	0.222
Tumor size (cm)			0.333
≤ 5.0	48	reference	
> 5.0	53		0.304
unknown	87		0.836
Positive pelvic node		--	0.170
Hemoglobin < 12 g/dL		--	0.525
Smoking		--	0.484
Field size (< 18 cm)		--	0.959
Pretreatment CEA level (ng/mL)			0.005
< 5	149	reference	
5-10	16	1.007 (0.232-4.365)	0.993
≥ 10	23	4.206 (1.801-9.821)	0.001
Pretreatment SCC-Ag level (ng/mL)			< 0.001
< 10	128	reference	
10-40	42	2.617 (1.180-5.804)	0.018
≥ 40	18	6.851 (2.722-16.935)	< 0.001

*Abbreviations*: CI = confidence interval; SCC-Ag = squamous cell carcinoma antigen; CEA = Carcinoembryonic antigen

**Table 3:** Multivariate analysis of PALN recurrence using CEA levels integrated into SCC-Ag level < 10 ng/mL

Parameters	Number	Hazard ratio (95% CI)	*p *value
Age		--	0.323
< 50	58	reference	
50-60	59	--	0.155
> 60	71	--	0.233
Stage		--	0.609
I	22	reference	
II	145	--	0.629
III-IVA	41	--	0.372
Parametrial score		--	0.121
0	36	reference	
1-3	103	--	0.111
4-6	49	--	0.040
Tumor size			0.651
≤ 5.0	48	reference	
> 5.0	53	--	0.517
unknown	87	--	0.864
Positive pelvic node		--	0.075
Hemoglobin < 12 g/dL		--	0.303
Smoking		--	0.528
Field size (< 18 cm)		--	0.874
Pretreatment tumor marker (ng/mL)			< 0.001
SCC-Ag < 10 and CEA < 10	113	reference	
SCC-Ag < 10 and CEA ≥ 10	15	8.838 (3.199-24.419)	< 0.001
SCC-Ag 10-40	42	4.246 (1.706-10.569)	0.002
SCC-Ag ≥ 40	18	12.551 (4.811-32.747)	< 0.001

*Abbreviations*: CI = confidence interval; SCC-Ag = squamous cell carcinoma antigen; CEA = Carcinoembryonic antigen

### PALN recurrence rates in relation to tumor marker combinations

**Figure 3:**
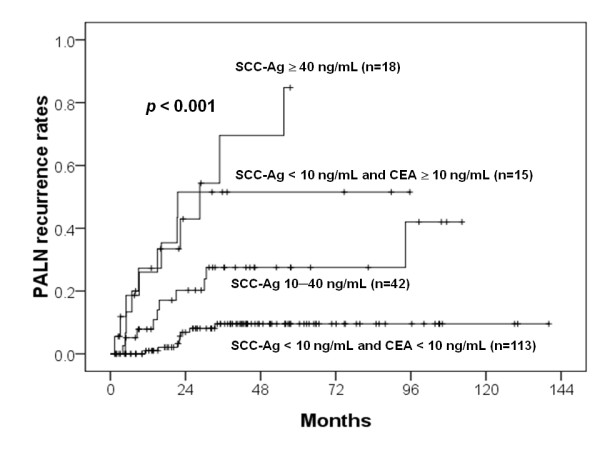
**PALN recurrent rates for various combinations of pretreatment of CEA and SCC-Ag levels**.

Patients with SCC-Ag levels ≥ 40 ng/mL, both CEA ≥ 10 ng/mL and SCC-Ag levels < 10 ng/mL, SCC-Ag levels of 10-40 ng/mL, and SCC-Ag levels of < 10 ng/mL had a 5-year PALN recurrence rate of 84.8%, 51.5%, 27.5%, and 9.6% (*p *< 0.001) (Figure 3), respectively. Hence, we categorized patients with SCC-Ag levels ≥ 40 ng/mL or both CEA ≥ 10 ng/mL and SCC-Ag levels < 10 ng/mL as the high-risk group (n = 33), in which the 5-year PALN recurrence rate was 66.5%. Patients with SCC-Ag levels of 10-40 ng/mL and SCC-Ag levels of < 10 ng/mL made up the intermediate- (n = 42) and low-risk group (n = 113), respectively.

## Discussion

CCRT improves the treatment outcome of locally advanced cervical cancer [1,2]. Morris et al. conducted a randomized trial to compare whole pelvic CCRT with extended field radiotherapy (EFRT) alone [1]. They found that CCRT improved overall survival and disease-free survival, as well as decreased locoregional and distant failure to a greater extent than EFRT. However, the 5-year recurrence rate of para-aortic lymph node (PALN) was 7% and 4% (*p *= 0.15) in the pelvic CCRT and EFRT groups in the updated data of the same study [10], respectively. Eifel et al. thought that the decrease of distant failure was attributable to a decrease of secondary spread from uncontrolled pelvic disease [10]. In our prior study, pelvic CCRT did not decrease PALN recurrence to a greater extent than pelvic RT alone [3]. Hence, cisplatin and 5-FU plus pelvic RT may not directly eradicate PALN recurrence. To eliminate PALN micrometastasis and recurrence, more aggressive treatments such as para-aortic lymphadenoectomy, prophylactic PALN irradiation, or additional chemotherapy should be considered in future clinical trials. Risk stratification is helpful to establish the eligibility of patients for the clinical trial.

PALN recurrence after definitive radiotherapy with or without concurrent chemotherapy is not uncommon in clinical practice. Niibe et al. proposed the concept of oligorecurrence and compared with oligometastasis [11]. PALN recurrence may be presented as either oligorecurrence or oligometastasis. Isolated PALN recurrence is classed as oligorecurrence. In this field, from Japan, a large population study (n = 3137) demonstrated that the incidence of isolated PALN recurrence was 2.1% [12]. The mean pretreatment SCC-Ag level was 17.3 ng/mL [12] that is similar to 16.7 ng/mL of present study. The correlation (r = 0.653; p = 0.002) between pretreatment and recurrence SCC-Ag level also confirmed the study of Niibe et al. [12]. The 5-year survival is around 30% for this type of oligorecurrence in our systematic review [3]. Non-isolated PALN recurrence often presents as oligometastasis that primary tumor is not controlled or with concurrent distant metastasis. The outcome is poor from our prior [3] and present results.

Whereas Eifel et al. reported a 5-year PALN recurrence of 7% following pelvic CCRT, our previous report [3] revealed a 5-year actuarial rate of 14%. In the present study, focusing on patients undergoing CCRT, the corresponding rate was 23.8%. Yeung *et al. *reported a PALN relapse rate of 15.3% in Stage IIIB/IVA patients [13]. Variations of PALN recurrence rates may depend on many factors related to physicians and patients. An intensive follow-up schedule could detect a higher rate of PALN recurrence [14]. Because the diagnosis is based on CT, MRI, or PET, unlike chest x-ray, these examinations are more expensive and cost-effectiveness is questionable. Many physicians do not perform intensive CT, MRI, or PET examinations at follow-up if normal tumor markers or no recurrent symptoms/signs are noted. The diagnosis of PALN recurrence may be delayed if tumor markers are not evaluated during routine follow-up. Once a patient shows clinically relevant failures, such as local failure, lung metastasis, or supraclavicular LN metastasis, the examination of PALN metastasis may be omitted and replaced with palliative care. Hence, the real incidence of PALN recurrence may be greater than the present data reflects. The distribution of poor prognostic factors in patients may be related to the incidence of PALN recurrence. The aim of the current study was to identify the risk factors.

We first identified CEA levels ≥ 10 ng/mL in patients with SCC-Ag levels < 10 ng/mL as a high-risk factor. Traditionally, SCC-Ag levels < 10 ng/mL is thought to be a low-risk factor [4]. The percentage of CEA levels ≥ 10 ng/mL was as small as 9.9% in patients with SCC-Ag levels < 10 ng/mL. This subgroup is easily overlooked because no study has demonstrated the role of CEA in PALN recurrence for squamous cell carcinoma of cervix. The onset of PALN recurrence was rapid and 2-year actuarial rate was high as 50%. Hence, CEA levels may be considered as an indicator of occult PALN metastasis in this subgroup. In addition, SCC-Ag levels ≥ 40 ng/mL was the other major risk factor of PALN recurrence. Based on HR and PALN recurrent rates, patients with SCC-Ag levels ≥ 40 ng/mL and CEA levels ≥ 10 ng/mL/SCC-Ag levels < 10 ng/mL were assigned to the high-risk group. Patients with SCC-Ag levels between 10 and 40 ng/mL made up the intermediate-risk group. Patients with both SCC-Ag and CEA levels < 10 ng/mL made up the low-risk group. We strongly suggest para-aortic lymphadenoectomy or PET-CT examination for planning the treatment of radiotherapy in intermediate- to high-risk patients. If isolated PALN metastasis is detected at the initial diagnosis of cervical cancer, the RT field should be extended to PALN lesions. Furthermore, PET-CT examination is suggested for patients with isolated PALN recurrence diagnosed with a CT scan to exclude another distant metastasis.

Some patients with occult PALN micrometastasis are at risk for PALN recurrence if they undergo pelvic CCRT for CT-negative PALN metastasis. Hence, para-aortic lymphadenoectomy can confirm subclinical PALN micrometastasis. Minimal complications are acceptable in modern laparoscopic technique providing that CCRT is not delayed [15,16]. A large series study (n = 253) [17] reports that for 17.9% of patients with pathology-confirmed PALN metastasis, their metastases were not detected in the CT scan at initial diagnosis of cervical cancer. The false negative rate of CT detection [18] was 23% in patients with laparoscopic extraperitoneal para-aortic lymphadenectomy. Taken together, the false negative CT detection rates of around 20% are compatible with the current incidence of PALN recurrence (18.6%). This finding implies that PALN recurrence may result from suboptimal pelvic CCRT for PALN micrometastasis. There are some advantages of laparoscopic extraperitoneal para-aortic lymphadenectomy. For example, it can prevent intermediate- to high-risk patients from receiving suboptimal treatment for pelvic radiotherapy. In addition, some intermediate- to high-risk patients with true-negative PALN metastasis are exempt from PALN irradiation because small bowel, bone marrow and renal toxicity are the major concerns. Hence, we suggest that screening for PALN metastasis in high-risk patients should be a priority. If laparoscopic extraperitoneal para-aortic lymphadenoectomy cannot be performed before radiotherapy for certain reasons, the PET-CT scan can be used as the alternative approach to detect PALN metastasis because of its false negative rate of 8.4% [16]. Prophylactic PALN irradiation is preserved in high-risk patients without para-aortic lymphadenoectomy or PET-CT scan examination. IMRT is suggested for prophylactic PALN plus CCRT because of acceptable acute, early, and late toxicities [19].

The prognostic factors of isolated PALN recurrence were CEA and SCC-Ag levels ≥ 10 ng/mL at recurrence and CEA levels ≥ 10 ng/mL at initial diagnosis. Although small number of isolated PALN recurrence, marginally statistical significance, and no long-term follow-up were noted, it is reasonable for impact of high markers at isolated PALN recurrence on survival because high SCC-Ag/CEA levels may be related to occult distant organ metastasis. Hence, early detection of PALN recurrence is very important for outcome because early isolated PALN recurrence can be salvaged [20] and tumor marker levels at PALN recurrence affect survival rate [3,21]. Because 40% of patients belonged to the intermediate to high-risk groups in our analysis, we suggest that intensive follow-up planning, such as SCC-Ag, CEA, and CT scan, in intermediate- to high-risk patients is indispensable to the early detection of PALN recurrence and early salvage. The current analysis demonstrated that no elevation of tumor markers and no symptoms were noted in 20% of PALN recurrences. Hence, tumor marker testing is not the only way to screen for PALN recurrence. Routine CT scan examination should be scheduled at follow-up.

Interestingly, initial CEA but not SCC-Ag levels could predict overall survival following isolated PALN recurrence. CEA is involved in brain metastasis of lung cancer [22] and liver metastasis of gastric cancer [23]. Moreno García et al. noted that the pretreatment CEA level was a prognostic factor of DFS, complete pathologic responses, and recurrences [24]. In Dukes' C2 rectal cancer, hepatic and local recurrence rates were higher in the patients with > 10 ng/mL of CEA than were those in the patients with < or = 5 ng/mL of CEA [25]. Hostetter et al. used a recombined CEA injection in mice and noted CEA-enhanced liver metastasis of colon cancer cells [26]. Hence, CEA may be a marker of PALN micrometastasis before CCRT for cervical cancer.

This study has strengths and some limitations. This is the first study to investigate the role of CEA and SCC-Ag levels in PALN recurrence following pelvic CCRT. We identify 3 risk groups and suggest intensive survey in intermediate- to high-risk patients. Patients with both CEA ≥ 10 ng/mL and SCC-Ag levels < 10 ng/mL should be managed with caution because they are at high risk for PALN recurrence. Compared with our prior study [3], this study is specific to patients undergoing CCRT and identifies CEA as a significant risk factor. We did not perform biopsy for tissue proof of isolated PALN recurrence, and only 4 patients had PET-CT examination, which was limited by the health insurance in our country. However, the outcome of isolated PALN recurrence was compatible with other studies [5,20,27]. In 227 patients, we excluded 39 patients without CT scan follow-up. Hence, the PALN recurrent rate may be overestimated because these 36 patients had low SCC-Ag levels (data not shown).

In conclusion, in addition to SCC-Ag levels, CEA is another important risk factor for PALN recurrence, especially in patients with SCC-Ag levels < 10 ng/mL. In those patients or patients with SCC-Ag levels ≥ 10 ng/mL, laparoscopic extraperitoneal para-aortic lymphadenectomy, PET-CT examination, or prophylactic PALN irradiation for radiotherapy planning should be considered first. Comprehensive evaluations before and after radiotherapy should be carried out. We suggest that clinical trials for aggressive detection and management of PALN metastasis are needed for the improvement of treatment outcome in these patients.

## Competing interests

The authors declare that they have no competing interests.

## Authors' contributions

EYH and YJH participated in design and coordination of study. EYH, YJ H, CCC, HL, CJW, LMS, CWT, CCT, YCO, HCF, HCC, HCH, and CYW participated in data acquisition. EYH and YJH contributed to the statistical analysis. EYH, YJH, CCW, CCC, and HL contributed to interpretation of data. EYH and YJH drafted the article and all other authors helped and finally approved the final manuscript.
